# Demand and willingness-to-pay for bed nets in Tanzania: results from a choice experiment

**DOI:** 10.1186/s12936-017-1929-2

**Published:** 2017-07-14

**Authors:** Chris D. Gingrich, Emily Ricotta, Amos Kahwa, Catherine Kahabuka, Hannah Koenker

**Affiliations:** 1 Eastern Mennonite University, 1200 Park Road, Harrisonburg, VA 22802 USA; 2 Johns Hopkins Center for Communication Programs, Baltimore, USA; 3 National Institutes of Health, Bethesda, USA; 4 National Institute for Medical Research, Muhimbili Medical Research Centre, Dar es Salaam, Tanzania; 5 CSK Research Solutions Ltd, Dar es Salaam, Tanzania

## Abstract

**Background:**

Universal coverage campaigns for long-lasting insecticide-treated nets do not always reach the goal of one net for every two household members, and even when ownership of at least one net per household is high, many households may not own enough nets. The retail market provides these households options for replacing or increasing the number of nets they own with products that best fit their needs since a variety of net shapes, sizes, and colours are available. Hence, it is important to understand the factors affecting private net demand. This study explores private demand for nets in Tanzania using a discrete choice experiment. The experiment provides participants the option to buy nets with their own money, and thus should prove more accurate than a hypothetical survey of net preferences.

**Results:**

Nearly 800 participants sampled in two regions showed an overall strong demand for nets, with 40% choosing to buy a net across all seven combinations of net prices and characteristics such as size, shape, and insecticide treatment. Only 8% of all participants chose not to buy a single net. A key factor influencing demand was whether a participant’s household currently owned sufficient nets for all members, with rural participants showing lower net coverage and greater demand than urban participants. Both poor and less poor households showed strong evidence of making purchase decisions based on more than price alone. Mean willingness-to-pay values for a net started at US$1.10 and grew by US$0.50–1.40 for various attributes such as rectangular shape, large size, and insecticide treatment. The impact of price on demand was negative but small, with elasticity values between −0.25 and −0.45.

**Conclusions:**

The results suggest that private demand for nets in Tanzania could potentially supplement future coverage campaigns. Net manufacturers and retailers should advertise and promote consumers’ preferred net attributes to improve sales and further expand net access and coverage. To overcome household liquidity concerns and best replicate the experiment results, policy makers should consider making credit available for interested buyers.

## Background

Since 2008, the World Health Organization (WHO) has recommended universal coverage with insecticide-treated bed nets (ITNs) to reduce malaria morbidity and mortality. Universal coverage campaigns aim to provide one ITN for every two people in regions of high malaria transmission [1]. Typically occurring every 3 years, these campaigns generally achieve high levels of ITN ownership and access [2]. However, even when ownership of at least one ITN is high, many households may not own enough ITNs for all family members. The retail market, which offers a variety of net shapes, sizes and colours, provides these households with options for replacing or increasing the number of ITNs they own with products that best fit their needs.

It is important to understand the factors affecting private net demand since purchased nets offer families a way to replace old nets or supplement public-sector distributions of long-lasting insecticide-treated nets (LLINs). Moreover, as policy makers are currently planning long-term national distribution strategies for bed nets, information such as price elasticities and willingness-to-pay (WTP) values should prove vital for designing future distribution schemes and understanding how markets can contribute to filling universal coverage gaps. One means of discerning this information, particularly when ample market data are lacking, is the use of experimental methods. Multiple techniques exist for researchers wanting to apply experimental methods to consumer preference questions. Auction exercises represent one commonly used approach, where participants gather in a group and record their maximum WTP for a product [3, 4]. In turn, the high or second highest bidder purchases the product with their own funds. While this technique produces a complete demand curve, showing price and quantity combinations for the group, its implementation presents special challenges, especially in a developing country. First, it requires that a large group of people concurrently complete the exercise. Second, participants must state their preferences in a manner different from their normal shopping experience. Rather than choose to buy a product at a given asking price, they must state their maximum bid price (i.e., WTP). Alfnes et al. argue that this concept is often difficult for participants to follow and implement [5]. Fortunately, a choice experiment or discrete choice experiment (DCE) circumvents these problems yet still yields an experiential derivation of consumer preferences. A DCE involves presenting two or more products (or other decision such as workplace scenarios) before a participant and asking which product she prefers at fixed prices [6]. A DCE thus presents an attractive means to gauge consumer preferences and WTP for bed nets in Tanzania. The bed net market in Tanzania has experienced significant demand and supply shocks since a study of consumer demand in 2007, namely the availability of pre-treated LLINs and a proliferation of free, mass distribution campaigns [7]. There are no known market data available since then. Tanzania’s bed net market features several kinds of available nets (untreated vs insecticide-treated, small vs large, rectangular vs conical) and consumers are generally familiar with these various attributes. This study explores private demand for bed nets in Tanzania using a DCE, with a special focus on WTP for various net types.

## Methods

Discrete choice experiments are best known for eliciting WTP values for products with no available market data, and particularly WTP values for various product attributes [6]. Other auction-type experiments do not provide comparable results. While DCEs may ask participants to state their hypothetical preferences among various products, it is also possible that DCE participants make non-hypothetical or ‘binding’ purchase decisions with their own money. When participants know their stated preferences imply binding decisions, the results will more likely reflect their true preferences. Existing research confirms a significant gap between hypothetical preferences and preferences obtained from binding experiments [8]. In particular, WTP estimates obtained from hypothetical DCEs typically show an upward bias.

The theoretical framework behind a DCE is well established [6, 9]. In short, when facing a choice between two goods, good A and B or neither, the participant chooses good A if its expected utility exceeds that of either good B or choosing nothing. Assuming that utility is a random linear function that depends on product attributes, it is straightforward to derive the probability that a participant chooses good A at various prices (i.e., a demand curve), demand elasticities and WTP values for the product attributes.

### Sample description

To obtain the sample DCE data for bed nets, this study targeted parents of schoolchildren via invitation letters sent home with students. A key advantage of this particular sample is that the school provided a central location to conduct the DCE and thus prevented field staff from carrying dozens of bed nets during door-to-door interviews. Ruvuma and Mwanza regions were chosen for the study sites, primarily because they both provided significant diversity with respect to urban and rural populations and were mid-tier in income rank. In addition, free LLIN distribution had been implemented more recently in most other regions and the assumption was that recipient households would show little interest in buying additional nets, regardless of their actual demand preferences. Mwanza region borders Lake Victoria in northern Tanzania. As of 2012, it ranked 14th in income per capita among 21 mainland regions at 900,000 TSH (approximately US$400) [10]. Ruvuma region lies at Tanzania’s southwest corner. It has a per capita income over 1,200,000 TSH (US$550), placing it fourth among mainland regions. National trends suggest a higher poverty incidence in rural areas (33% poverty rate for rural vs 22% for all urban outside Dar es Salaam), and there is no reason to suspect Mwanza and Ruvuma show a different pattern.

Both regions have a recent history of progressive bed net programmes, first under a national subsidized voucher scheme for pregnant women and infants from 2004 to 2014, a free under-five coverage campaign between 2008 and 2010, and free universal coverage campaigns in 2010, 2011 and 2015 [11–14]. Fieldwork for this study occurred 12 months after the 2015 campaign in Mwanza (National Malaria Control Programme, pers. comm., [13]). Ruvuma implemented its universal coverage campaign in November 2010, and then began annual rounds of large-scale school distribution in July 2013, August 2014 and August 2015 (National Malaria Control Programme, pers. comm., [11]). Except for the most recent school distributions in Ruvuma, each region’s history with nets and net distribution schemes is comparable. One urban and one rural district for each region were used as data collection sites, namely Nyamagana and Magu districts in Mwanza and Songea and Mbinga districts in Ruvuma.

Two school sites were utilized in each district. The specific schools were selected after consulting with local health department and education officials, who chose schools easily accessible by road yet also containing a broad mix of household income levels. Another criteria was the willingness of school administrators and head teachers to participate in the exercise. School administrators selected students from each grade, one to seven, and chose more students from larger sized grades. Moreover, they selected roughly an equal proportion of boys and girls and students from low- and high-income groups. To maximize participation, administrators prioritized students living closer to the schools and whose parents had a reputation of responding to meeting requests. The invitation letters stated the intent of the exercise and explained that participants would receive 10,000 TSH plus the opportunity to purchase a bed net at a discounted price between 2000 and 8000 TSH (US$1 = 2200 TSH). The prices originated from an informal survey of Dar es Salaam markets in October 2015 (8 months before the DCE data collection began) showing prices for both treated and untreated nets in the 8000–10,000 TSH range.

While the sample is not nationally representative of all households, it represents (non-randomly selected) households with school-age children in the two regions. Selection bias may have occurred at several stages of data collection, including the choice of district, the choice of school, and student selection to receive invitation letters. Additionally, participants possibly self-selected according to their potential interest in obtaining a net.

Field staff were recruited by CSK Research Solutions, Ltd, Dar es Salaam, Tanzania, and underwent 5 days’ training in both DCE and general research methods. Three authors were extensively involved with the training, which included role playing and mock experiments to ensure the staff clearly understood DCE theory and methods. Prior to data collection, including 2 days of piloting with 20 participants in Kinondoni district near Dar es Salaam, the National Institute for Medical Research (NIMR) in Tanzania and the Johns Hopkins School of Public Health Institutional Review Board granted full approval for the study.

Following Hensher et al., the minimum sample size for viable choice experiments is roughly 50 observations for each selected option (i.e., net A, net B, or neither net) [15]. Hence, treating all four sites separately and allowing that participants would likely not choose each option equally, 200 participants for each site became the desired sample size. In total, 961 invitation letters were distributed for an overall potential participant yield of 83%, with individual school yields ranging from 61 to 97%. Field staff reported that 100% of potential participants consented to complete the preliminary survey and DCE. All interviews occurred between 23 May and 14 June, 2016. Two teams of field staff (two persons each) along with one supervisor worked concurrently in Mwanza and Ruvuma. Roughly 200 participants completed the DCE in each district, for 801 total participants.

### Demographic and ideation information

Prior to completing the DCE portion of the interview, participants answered a series of questions covering their household demographics, asset ownership (including bed nets), understanding and perceptions of malaria, and familiarity with and use of bed nets. Measurement of these cognitive, emotional and social factors is defined as ‘ideation’ [16, 17]. In this study, the cognitive dimension includes attitudes, knowledge about malaria and its prevention, perception of severity and susceptibility to malaria, belief that nets are an effective malaria prevention tool (also known as response efficacy), and knowledge about where to purchase additional nets. The emotional dimension focuses on perceived self-efficacy to use a net to protect against malaria, and belief in one’s ability to obtain enough nets for the household. For the social interaction dimension, the constructs include perceived social norms (whether other households in the community are regularly using nets), and interpersonal communication about decision making for net purchasing and use. The assumption behind the ideation model is that the more of these ideational variables a person has, it is more likely they will behave in a certain fashion.

Questions on perceptions of malaria and bed nets were constructed using a four-point Likert scale, ranging from ‘definitely could not’ to ‘definitely could’, or from ‘strongly disagree’ to ‘strongly agree’. Variables were re-coded −2 to +2, with factor analysis applied to variables grouped into ideational constructs (e.g., perceived susceptibility and perceived severity). The results were split into high/low summary categories by noting whether each household’s average category value was greater or less than zero. A partial list of ideation questions and responses appears in the next section. A full list is available on request.

### DCE design

The DCE aimed to measure consumer preferences for bed nets that are readily available in the Tanzanian marketplace. Hence, nets possibly known to households only through non-market channels, such as free distribution campaigns (e.g., PermaNet brand LLINs), were not considered. In addition, not all nets were available in every possible size and shape combination.

Table 1 summarizes the various bed net attributes and levels examined in the DCE. Both the treated and untreated nets, Olyset and Safinet respectively, are manufactured by A to Z Textile Mills, Ltd, in Arusha, Tanzania and available in most local markets. Packages were clearly marked with their brand name and the Olyset nets are also marked as insecticide treated. Field staff also explained the treated/untreated attribute to participants.

**Table 1:** Summary of net attributes examined in the DCE

Attribute and level				Available colour^a^
Treated (T) or untreated (U)	Shape	Size (feet)	Price (000 TSH)	
T (Olyset brand)	Rectangular	4 × 6	2, 4, 6, 8	Aqua blue
T (Olyset brand)	Rectangular	6 × 6	2, 4, 6, 8	Navy blue and white^b^
U (Safinet brand)	Rectangular	4 × 6; 6 × 6	2, 4, 6, 8	White
U (Safinet brand)	Conical	3.5 × 6^c;^ 6 × 6	2, 4, 6, 8	White

^a^Colour is not an attribute examined in the DCE, though it possibly influenced participants’ decisions. Field staff did not mention colour during the DCE but it was visible through the clear packaging

^b^Most large Olyset nets used in Mwanza region were white but a few navy blue nets were also included. All large Olyset nets in Ruvuma were navy blue

^c^In terms of size attribute, level 3.5 × 6 is considered identical to level 4 × 6. Both 3.5 × 6 and 4 × 6 are listed as ‘small’ and 6 × 6 are listed as ‘large’

A complete matrix of all attributes and levels in Table 1 yielded 24 different ‘A vs B’ net combinations to consider. Obviously, this presented too many options for any one person to meaningfully consider during a single interview so the following process reduced the number of questions to each participant: first, a fractional design reduced the total DCE scenarios to 14 [18]. Second, the 14 questions were separated into two equally sized ‘blocks’, where each participant was randomly assigned to complete either Block 1 or Block 2. The resulting set of net comparisons appears in Table 2.

**Table 2:** Fractional factorial design used for the DCE

Block	Scenario number	Net A				Net B			
		Brand	Size	Shape	Price (TSH)	Brand	Size	Shape	Price (TSH)
1	1	Safinet	Large	Conical	2000	Olyset	Small	Rectangular	4000
1	2	Safinet	Large	Rectangular	8000	Safinet	Small	Rectangular	2000
1	3	Safinet	Small	Conical	4000	Safinet	Large	Conical	4000
1	4	Safinet	Large	Conical	6000	Safinet	Large	Conical	2000
1	5	Safinet	Small	Rectangular	6000	Olyset	Large	Rectangular	6000
1	6	Olyset	Small	Rectangular	4000	Safinet	Large	Rectangular	8000
1	7	Safinet	Large	Rectangular	4000	Olyset	Small	Rectangular	8000
2	1	Olyset	Large	Rectangular	2000	Safinet	Small	Conical	4000
2	2	Safinet	Small	Conical	8000	Safinet	Small	Rectangular	6000
2	3	Safinet	Small	Conical	2000	Safinet	large	Rectangular	4000
2	4	Olyset	Large	Rectangular	6000	Safinet	Large	Conical	6000
2	5	Safinet	Large	Conical	4000	Safinet	Small	Conical	2000
2	6	Safinet	Small	Rectangular	2000	Safinet	Small	Conical	8000
2	7	Olyset	Small	Rectangular	8000	Olyset	Large	Rectangular	2000

In addition to the randomly assigned blocks, each participant was randomly assigned one of two different groups to complete scenarios 1 through 7. Group 1 completed the scenarios in order 5, 2, 4, 7, 6, 1, 3 while Group 2 completed the scenarios in order 2, 5, 4, 6, 7, 3, 1, with each sequence generated using a random number generator without replacement. Both the block and order randomized assignments occurred automatically using a data management system on tablet computers.

To prepare participants for the DCE using nets, the field staff first presented two ‘warm up’ scenarios using two pieces of candy with similar choices (candy A, candy B, or neither A or B), each costing 50 or 100 TSH. Participants received 100 TSH before the exercise and after each participant stated her choices, a random card selection (1 or 2) identified the ‘binding’ scenario, followed by an actual purchase if applicable and change provided to the participant. The cards used were from a Rook^®^ game, which do not resemble traditional playing cards, and were intentionally chosen to avoid any negative connotations to gambling. Besides employing only two scenarios and a smaller cash payoff, these warm-up DCE exercises conformed exactly to the DCE using nets. For the subsequent net-based DCE, participants received 10,000 TSH before responding to all seven scenarios for the participants’ assigned block. At the end of the seven DCE scenarios with all choices recorded, the participant blindly chose a card between 1 and 7. The card identified the binding scenario, where the preferred net (if any) was purchased with the 10,000 TSH stipend and the remaining balance provided to the participant.

The procedure for estimating a demand curve for bed nets using DCE data requires treating each individual purchase decision as separate binary outcomes that depend on factors such as net attributes, price and the household’s socio-economic status. In other words, each DCE scenario for each participant yields three separate binary ‘observations’ for demand estimation: Choose net A, choose net B, and choose neither net A nor net B. The complete procedure, using conditional logit estimation, is well detailed in Aizaki, Aizaki et al., and Aizaki and Nishimura [18–20]. Equation (1) represents a generic form of bed net demand based on the DCE.

1 $${\text{Buy}} = {\text{f}}\left( {{\text{ASC}},{\text{ treatment}},{\text{ rectangular}},{\text{ large}},{\text{ price}},{\text{ LessPoor}}} \right).$$

A full description of each variable appears in “Results” section. Variable LessPoor, which indicates the top three socio-economic quintiles, enters into (1) in two ways: first as an interactive variable with ASC to capture any potential demand shifts due to socio-economic status and second, as an interactive variable with price to explore whether socio-economic status affects price elasticity.

## Results

### Household demographics and net ownership

All data were collected electronically and generally error-free. The DCE scenarios contained ‘did not answer’ responses for three participants and data for these individuals were removed. In addition, two participants were either troubled by the DCE exercise or could not grasp the concept (as determined by the field staff) and their answers were removed from the analysis. Thus, the final sample contained 796 observations. A brief summary of the participants appears in Table 3. Among all participants, 66% were female with 76% female in the urban districts and 56% female in the rural districts.

**Table 3:** Description of participants, percent (number), total n = 796

Description	Response				
Status within household	Head of household	Spouse of head	Other		Missing or did not answer
	49.0 (390)	39.4 (314)	11.4 (91)		0.1 (1)
Gender	Female	Male			Missing or did not answer
	66.3 (528)	33.4 (266)			0.3 (2)
Age, mean number of years	39.3				
Can the household head read and write?	Yes	No			Missing or did not answer
	91.8 (731)	7.7 (61)			0.5 (10)
Did the household head attended school?	Yes	No			Missing or did not answer
	93.5 (744)	6.2 (49)			0.4 (3)
Highest level of schooling, head of household	Primary	Secondary	Higher	Other	Missing or did not answer
	66.9 (498)	26.7 (199)	5.9 (44)	0.3 (2)	0.1 (1)
Who is responsible for purchasing nets for your household?	Self	Spouse	Other		Missing or did not answer
	72.4 (576)	17.7 (141)	9.3 (74)		0.7 (5)

Households in each district own significant quantities of nets, of which between one-third and one half, on average, were purchased (Table 4). Households in all districts show average net ownership at or above the generally accepted standard of one net per every two people, or a net ratio of 0.50 nets per resident [1]. Note, however, that rural Mwanza’s average net ratio (0.55) lies substantially below urban Mwanza (0.82) and standard deviations are large for all districts (0.24 to 0.45). Information regarding households’ net use or whether the nets were insecticide treated was not gathered.

**Table 4:** Household net ownership, by location

	Average number of nets owned per household (std dev)	Average number of owned nets that were purchased per household (std dev)	Average number of nets owned per household resident (std dev)
Mwanza urban	4.77 (2.60)	2.54 (1.87)	0.82 (0.45)
Ruvuma urban	3.47 (1.90)	1.48 (1.57)	0.63 (0.30)
Mwanza rural	3.79 (1.82)	1.16 (1.45)	0.55 (0.24)
Ruvuma rural	3.15 (1.80)	1.02 (1.32)	0.63 (0.35)

### Overall propensity to buy nets

Table 5 reveals participants’ overall propensity to buy nets, showing the number of times they choose to buy either net A or net B out of seven scenarios. Stated differently, participants in the ‘0’ category (7.8%) chose to buy no nets for any scenario and were guaranteed to take home 10,000 TSH. Participants in category ‘7’ (39.7%) chose to buy either net A or B for all seven scenarios and were guaranteed to receive a net regardless of the random card drawn. Participants in categories 1 through 6 found at least 1 scenario where they chose to purchase a net and in other scenario(s) preferred to keep the entire 10,000 TSH.

**Table 5:** Distribution of participants’ DCE choices

Number of times participant chose to buy either net A or net B (out of 7 maximum)	Frequency	
	Percent	Number
0	7.8	62
1	8.0	64
2	7.0	56
3	8.3	66
4	9.2	73
5	8.9	71
6	11.0	88
7	39.7	316
All	100.0	796

Results aggregated for both blocks combined across all locations

**Fig. 1:**
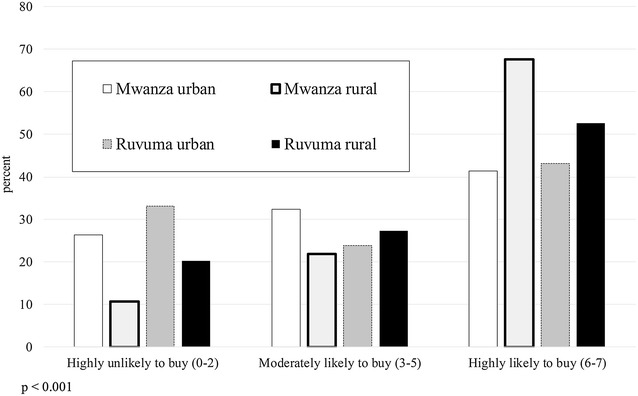
Propensity to buy a net (number of times purchased out of seven scenarios), by district

**Fig. 2:**
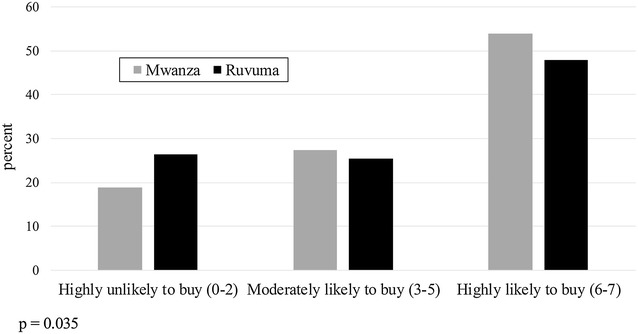
Propensity to buy a net (number of times purchased out of seven scenarios), by region

**Fig. 3:**
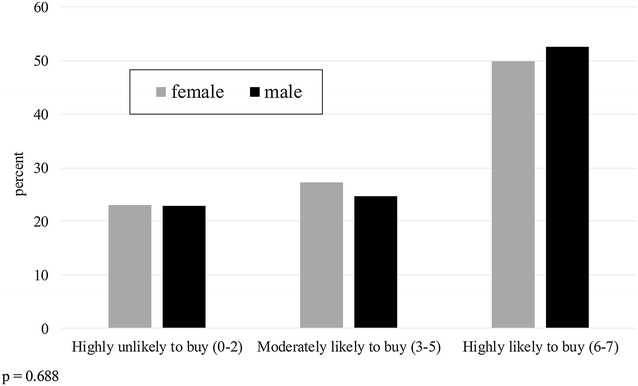
Propensity to buy a net (number of times purchased out of seven scenarios), by gender of participant

Figure 1 compares net buying behaviour across all four districts. The propensity to buy categories are condensed into three groups: Highly Unlikely to Buy (zero to two nets selected out of seven scenarios), Moderately Likely to Buy (three to five out of seven), and Highly Likely to Buy (six to seven out of seven). A Chi square test for independence between location and propensity to buy rejected this hypothesis (p < 0.001), with rural participants showing an overall higher propensity to buy than urban participants. A similar test for independence between propensity to buy and region shows participants from Mwanza with a higher propensity to buy than those from Ruvuma (Fig. 2; p = 0.035). The participant’s gender did not significantly affect their propensity to buy a net (Fig. 3; p = 0.69).

Principal components analysis was used to create socio-economic status indices based on each household’s ownership of various assets, sanitation and water access, cooking fuel type, and the head’s education level [21, 22]. Each household was then assigned a socio-economic quintile rank, with 74.9% of households from the two poorest quintiles located in rural districts. However, by separate Chi square test, socio-economic status and propensity to buy are independent (p = 0.489).

Two critical questions are whether participants understood the DCE exercise and whether it mimics actual marketplace behaviour. One way to explore these questions is to test whether participants behaved both rationally and consistently. The term ‘rational’ in this case implies that participants choose a lower price net (or no purchase) when two identical nets appear in the same scenario. Consistency suggests that participants who show preferences for a specific net attribute behave similarly across multiple scenarios. For example, Table 2 suggests that a rational participant would not choose net A for Block 1 Scenario 4 (since net A costs 4000 TSH more than identical net B), nor net A for Block 2 Scenario 7 (since net A costs 6000 TSH more for a small size net versus an otherwise identical large size net B). For Block 1 Scenario 4, 24.9% of participants chose net A and 19.2% chose net A for Block 2 Scenario 7.

Although both results show a surprising degree of ‘irrationality’, closer inspection reveals that some participants believe a low price net signals low quality to the consumer. Evidence that low price signals low quality appears in the ideation survey, which asks whether participants agree with the statement: “More expensive bed nets are more effective than less expensive or free bed nets”. Nearly half of the participants either strongly agreed (38.6%) or somewhat agreed (6.5%) with the statement. Conversely, 42.2% strongly disagreed and 8.2% somewhat disagreed with the statement. More revealing is that for Block 1 participants, a higher share of those agreeing with the statement chose net A for Scenario 4 compared to those not in agreement (33.6 vs 19.3%; p = 0.003). Similarly, a higher share of Block 2 participants who agreed with the statement chose net A for Scenario 7 (24.9 vs 13.9%; p = 0.011). Both findings suggest a strong association between the belief that more expensive bed nets are more effective and willingness to purchase a more expensive but identical net. The broad conclusion is that rationality prevailed throughout the sample, except for participants who believed that higher priced nets implied higher quality.

Regarding consistency, the first test examines Block 1, where the same participants willing to pay 6000 TSH more for a large net (net A) in Scenario 2 logically should not accept a small net (net A) for the same price as a large net in Scenario 3. In other words, a disproportionately large number of participants who choose A for Scenario 2 should also choose B for Scenario 3. A Chi square test for independence between Block 1, Scenario 2 and Scenario 3, confirms that a disproportionately large number of participants prefer a large net in both scenarios (p < 0.001). Similarly, Block 2 participants willing to pay 6000 TSH more for a conical net in Scenario 6 (B) should more likely pay 2000 TSH extra for a conical net in Scenario 2 (A). A Chi square test for independence confirms that a disproportionately large number of participants prefer a conical net in both scenarios (p < 0.001).

As seen in Table 6, bed net and malaria ideation indicators were generally high, with a strong positive belief about nets and their benefit for malaria prevention. Over 85% of participants knew where to buy a new net if they wanted to purchase one, and 78% of people felt capable of obtaining enough nets for their family. While a higher proportion of individuals had low perceived severity of malaria (59%), almost 81% felt that their family’s susceptibility to malaria was high. Over three-fourths (76%) of all participants could recall exposure to a malaria-related message within the past 6 months (via health clinic, radio, newspaper, etc.).

**Table 6:** Summary of bed net and malaria ideation questions (total n = 796)

Category (variable name), questions, and summary	Response, percent (n)					
Social norm of net use (*Socnorm*)	Hardly any	Less than half	More than half	Most	All	Do not know
“Generally, in how many households in your community do people sleep under a bed net?”	11.1 (88)	11.6 (92)	8.5 (68)	42.7 (340)	17.6 (140)	8.5 (68)
Perceived severity of malaria	Strongly disagree	Somewhat disagree	Somewhat agree	Strongly agree		Uncertain/did not answer
“I don’t worry about malaria because it can be easily treated”	24.0 (191)	12.3 (98)	14.2 (113)	49.2 (392)		0.3 (2)
“My children are so healthy that they would be able to recover from a case of malaria”	28.4 (226)	8.0 (64)	14.2 (113)	49.0 (390)		0.4 (3)
“Only weak children can die from malaria”	53.8 (428)	9.4 (75)	7.3 (58)	29.1 (232)		0.4 (3)
“When my child has a fever, I almost always worry that it might be malaria”	7.9 (63)	3.0 (24)	12.8 (102)	76.0 (605)		0.3 (2)
*Ideation summary*	Percent high (somewhat/strongly agree) = 40.5					
Perceived susceptibility to malaria (*Suscept*)	Strongly disagree	Somewhat disagree	Somewhat agree	Strongly agree		Uncertain/did not answer
“During the rainy season, I worry almost every day that someone in my family will get malaria”	9.5 (76)	4.6 (37)	14.3 (114)	71.2 (567)		0.3 (2)
“People only get malaria when there are lots of mosquitoes”	9.5 (78)	4.3 (34)	7.2 (57)	78.6 (626)		0.1 (1)
“Nearly every year, someone in this community gets a serious case of malaria”	10.4 (83)	4.0 (32)	12.1 (96)	73.0 (581)		0.5 (4)
“I cannot remember the last time someone I know became sick with malaria”	52.3 (416)	7.0 (56)	9.7 (77)	30.8 (245)		0.3 (2)
“I know people who have become dangerously sick with malaria”	13.8 (110)	4.0 (32)	11.2 (89)	70.9 (564)		0.1 (1)
“When my child has a fever, I almost always worry that it might be malaria”	7.9 (63)	3.0 (24)	12.8 (102)	76.0 (605)		0.3 (2)
*Ideation summary*	Percent high (somewhat/strongly agree) = 80.9					
Perceived ability to obtain enough nets (*Obtain*)	Definitely could not	Probably could not	Probably could	Definitely could		Uncertain/did not answer
“Obtain enough bed nets for all your children”	17.5 (139)	4.4 (35)	13.3 (106)	64.8 (516)		0.0 (0)
Know where to buy a net	Definitely could not	Probably could not	Probably could	Definitely could		Uncertain/did not answer
“Find a net seller nearby if I wanted to purchase one”	11.7 (93)	3.1 (25)	10.7 (85)	74.5 (593)		0.0 (0)
Price efficacy of nets	Strongly disagree	Somewhat disagree	Somewhat agree	Strongly agree		Uncertain/did not answer
“More expensive bed nets are more effective than less expensive or free bed nets”	42.2 (336)	8.2 (65)	6.5 (52)	28.6 (307)		4.5 (36)
Exposure to malaria messaging	Yes	No				Missing/did not answer
“In the past 6 months, have you seen or heard any messages about malaria [on TV or radio]?”	76.1 (606)	23.9 (190)				0.0 (0)

**Fig. 4:**
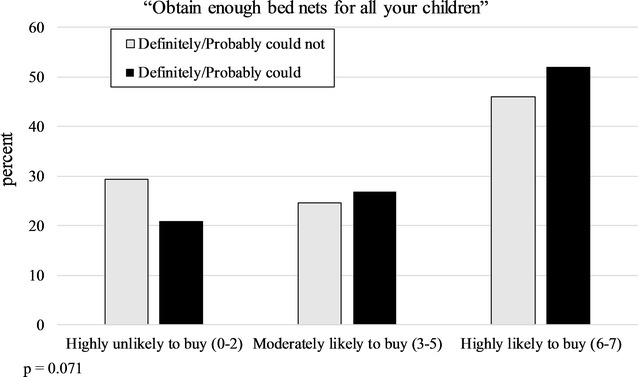
Propensity to buy a net (number of times purchased out of seven scenarios), by ideation variable *Obtain*

The malaria ideation and bed net variables show a mixed impact on participants’ propensity to buy a net. Variable *Obtain* is significantly and positively correlated with propensity to buy, though not to a large degree (p = 0.071; Fig. 4). However, variables *Suscept* and *Socnorm* are both statistically independent of propensity to buy (p = 0.999 and p = 0.234, respectively). Moreover, all three ideation variables are independent of the household’s urban versus rural location (p values between 0.371 and 0.864). Exposure to malaria messaging did not vary by region (p = 0.804) and was independent of propensity to buy (p = 0.359).

**Fig. 5:**
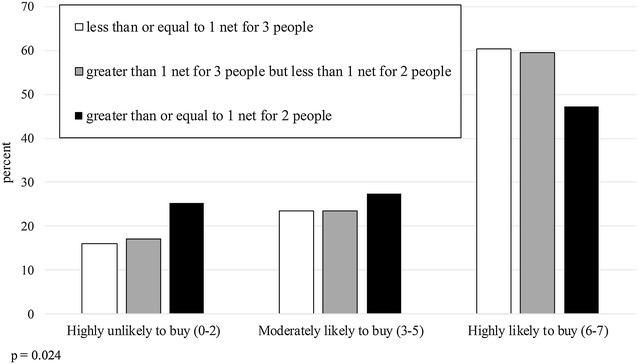
Propensity to buy a net (number of times purchased out of seven scenarios), by household net ratio

Whether households own sufficient nets to cover their inhabitants appears to affect their propensity to buy a net. Figure 5 plots participants’ propensity to buy a net against household net ratios (the number of nets owned per resident). In general, households’ likelihood of buying a net declines as the number of nets per person increases, suggesting that participants made their purchase decision, in part, based on their immediate need for a net (p = 0.024). Net ratio varies by location, with 67.9% of rural households owning at least one net per two people compared to 78.5% of urban households (p < 0.001). Moreover, among households with sub-standard net ratios, rural locations show a higher, though non-significant, mean number of pregnant women plus children under 5 years old than urban locations (1.63 vs 1.36 per household, respectively; p = 0.111). Hence, lower net ratios and greater vulnerability to malaria at least partly explain rural households’ greater propensity to buy.

### Demand and WTP for nets

Definitions for all Eq. (1) variables appear in Table 7, with corresponding regression estimates in Table 8. The restricted estimates reflect omission of the ASC: LessPoor interactive variable which is not statistically significant. In the restricted model, all variables are significant at the 99% confidence level.

**Table 7:** Variables used to estimate bed net demand, Eq. (1)

Variable	Description
Buy	Dependent binary variable = 1 if the individual acted on this choice or = 0 if they did nothing for the specific choice
ASC	Binary variable = 1 denoting either net A or net B, otherwise = 0 for neither net A nor net B
Treatment	Binary variable = 1 if net is brand Olyset (i.e., a treated net)
Rectangular	Binary variable = 1 if net is rectangular shape
Large	Binary variable = 1 if net is large (6 × 6) size
Price	Price of 2000; 4000; 6000; or 8000 TSH
LessPoor	Binary variable = 1 if participant’s household belongs in the upper three socioeconomic quintiles.

**Table 8:** Conditional logit estimate of the DCE demand model (n = 796)

Variable	Unrestricted		Restricted	
	Coefficient	p value	Coefficient	p value
ASC	0.359	<0.001	0.293	<0.001
Treatment	0.256	<0.001	0.255	<0.001
Large	0.283	<0.001	0.284	<0.001
Rectangular	0.174	0.001	0.175	0.001
Price	−0.00009	<0.001	−0.000084	<0.001
ASC: Lesspoor (interactive variable)	−0.109	0.272	–	–
Price: LessPoor (interactive variable)	−0.000057	0.002	−0.000073	<0.001
Rho squared goodness of fit indicator (0–1)	0.031		0.031	

The estimated DCE model generally shows expected results. Significant and positive estimates for coefficients ‘Treatment’, ‘Large’ and ‘Rectangular’ suggest that most participants were willing to pay extra for these specific net attributes (amounts discussed below). Price negatively affects net purchases. While socio-economic status does not significantly affect overall net demand, relatively wealthy households show a larger (negative) impact of price on their purchases.

**Fig. 6:**
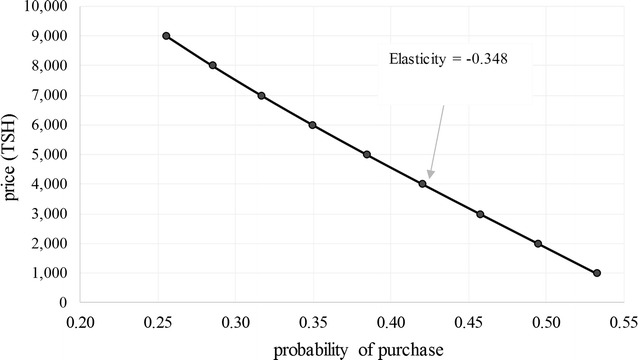
Demand curve for a large, rectangular, Olyset net (less poor household)

The estimated coefficients also yield purchase probabilities, price elasticities of demand and WTP values [9, 23, 24]. For example, Table 9 shows purchase probabilities and price elasticities for two different net types (a large rectangular Olyset net and a small conical Safinet net, both priced at 4000 TSH), by socio-economic status. Purchase probabilities range from 0.26 to 0.44. Varying the price of a large, square Olyset net from 1000 to 9000 TSH yields a complete demand curve (Fig. 6). In all cases, demand is highly inelastic, with price elasticities ranging from −0.21 to −0.44.

**Table 9:** Purchase probabilities (and price elasticities) for two different net types, by socio-economic status

	Large, rectangular Olyset at 4000 TSH^*^	Small, conical Safinet at 4000 TSH^*^
Less poor (top three quintiles)	0.421 (−0.348)	0.263 (−0.442)
Poor (bottom two quintiles)	0.444 (−0.207)	0.282 (−0.268)

*Comparison net (i.e., net B) is a small rectangular Olyset net priced at 4000 TSH

The final result from the demand model is mean WTP estimates (Table 10). The WTP estimates range for a small, conical, untreated net (the ‘base’ net identified by the ASC variable) from 2393 TSH (less poor households) to 3850 TSH (poorer households). For attribute ‘upgrades’, including insecticide treatment, large size and rectangular shape, mean WTP varies from 1161 to 3041 TSH, with shape showing the smallest WTP and size the largest WTP. Households from the bottom quintiles show WTP values for upgrades that are generally 700–1000 TSH higher than for less poor households.

**Table 10:** Mean WTP estimates (in TSH) for net attributes, by socio-economic status

Variable/attribute	Poor (bottom two quintiles)	Less poor (top three quintiles)	Interpretation
ASC	3850	2393	WTP for a small, conical, untreated net
Treatment	2742	1704	Additional WTP for a treated net
Large	3041	1890	Additional WTP for a large net
Rectangular	1868	1161	Additional WTP for a rectangular net

## Discussion

The DCE results and accompanying survey questions indicate a high degree of awareness among Tanzanians regarding the causes and prevention of malaria, and an overall strong demand for bed nets. Moreover, the typical Tanzanian net buyer carefully weighs factors such as shape, size and treatment/material, in addition to price, during her purchase decision. Such factors are important enough that she is willing to pay (roughly 2000 TSH) for the relevant upgrade. It is in net manufacturers’ and retailers’ best interest to promote such attributes. Further research using focus groups, etc. should be conducted to confirm that they match the desired upgrades found here (rectangular shape, large size, treated/polyethylene). Retailers and policy makers should also examine constraints on buyers stemming from liquidity shortages, provide consumer education, and review tax and tariff policies with the goal of shifting consumers from untreated nets to LLINs. Fortunately, since the findings show households with a moderate willingness to pay for higher-priced treated nets, it should be relatively easy to reinforce the importance of insecticide treatment in both public and private marketing campaigns. Overall, retail sales, in conjunction with large-scale, public-sector distributions and as part of a larger bed net strategy, can help fill gaps in household net ownership.

The strong demand results may partly reflect priming influences. In general, priming refers to changes in consumer behaviour that occur due to conscious or subconscious exposure to a related idea, theme or image. Recall that respondents faced a total of 27 bed net and malaria ideation-related questions before completing the DCE. The evidence in the literature for similar priming effects on consumers is quite strong [25–27]. Mandel and Johnson describe the type of positive demand shift that may have occurred here as semantic or conceptual priming [25]. Unfortunately, the study did not feature a control group that completed the DCE scenarios before answering the malaria and bed net questions.

While price elasticities of demand are quite low (less than −0.50), they resemble results from a randomized trial on ITN demand in Madagascar [28]. They also suggest that further price reductions beyond the values used in the study (2000–8000 TSH) would only minimally improve net coverage. Price elasticities at the full retail price should be larger since higher prices would mean that each potential purchase comprises a greater share of a household’s income. In reality, however, price elasticities for less poor households were slightly higher (though still inelastic) than for households in the two poorest quintiles.

Recall that the share of ‘irrational’ participants, those choosing to buy high-priced nets over equivalent low-priced nets, was not trivial (19–25%). These shares fell slightly after excluding participants who agreed with a survey statement that “low-priced nets are inferior to high-priced nets” (14–19%). Zeithaml argues that the relationship between price and perceived quality is complex and thus unlikely to be fully captured by a single survey question [29]. Hence, other participants may have made decisions assuming that low price signals low quality even though they did not explicitly agree with the survey statement. Zeithaml also argues that “low price–low quality” perceptions will be strongest when price differences are large, as they are here (200–300% price differences between net A and net B). The marketing research literature further suggests that this perception can be pervasive, affect consumer decisions, and is commonly found for side-by-side product comparisons [30]. Widespread presence of counterfeit goods in Tanzanian markets may have also caused participants to subconsciously follow this perception even though not explicitly stated.

Despite the overall strong demand for nets in Tanzania, a word of caution pertains to the poorest households still unable or unwilling to buy nets. The DCE results for elasticity, WTP, etc. only refer to the sample mean, suggesting that outlier households (even within the poor vs less poor categories) may show behaviours very different than those reported here. Moreover, the sample population is not nationally representative and there may be poorer sub-groups not captured in the data. Specific non-market delivery channels for these groups should be explored as warranted, which lies beyond the scope of this study.

A second caveat concerns how this study’s conclusion of strong net demand might apply immediately following a mass distribution campaign. The above results predict and one study of a prior campaign in Tanzania describes how private sales will decline once households’ short term needs for nets becomes saturated [31]. A strong demand for nets cannot be expected to continue without pause immediately following any future mass delivery campaigns. However, this demand will be contingent on a mass campaign’s ability to fully supply all households. For example, a recent survey found that immediately following the 2015 universal coverage campaign in Mwanza, 90.3% of all households in the region owned at least one treated net, while only 57.1% of households had one ITN for every two household members [32]. In Ruvuma, where a universal coverage campaign was not conducted but school distribution had occurred annually since 2013, 66.1% of households owned at least one ITN, and only 36.6% owned one ITN for every two people. It is extraordinarily difficult for distribution campaigns of treated nets to reach greater than 70% of households with one net for every two people. Overall demand is likely to be lower after a mass campaign but should not reach zero, given the inevitable gaps in household net ownership. Similarly in this study, rural participants’ overall higher propensity to buy likely stems from their lower overall net access per capita rather than income-related factors,

Three critical questions remain regarding the overall DCE design. First, it is unclear how the cash stipend (endowment) might have affected participant behaviour. One previous DCE study suggests a small positive impact on purchases, provided the endowment does not greatly exceed the market value of the good in the experiment [33]. For this study, the stipend/endowment was needed so that participants would have cash available to buy a net if they chose that option. With no stipend the demand results would be biased downward due to cash/liquidity-constrained participants with a strong affinity for nets. For example, a recent randomized trial regarding the impact of micro-loans on unsubsidized bed net purchases in India showed an overall purchase rate of 52% with available credit versus 10.8% without credit [34]. Stated differently, the DCE should accurately measure demand if short term, zero-interest loans are readily available to potential net buyers.

Second, a bed net is best conceptualized as a durable or investment good, where a potential buyer has several months to consider a potential purchase (e.g., in the case of a pregnancy) and the net remains functional for several years. However, the DCE compresses this investment decision into an immediate consumption decision, with no time allowed to fully consider product information, mosquito control alternatives (such as coils or environmental improvements), etc. It is unclear how this change affected participants’ behaviour in the DCE.

Finally, it is unclear the extent that selection bias, for the districts, schools or student invitations, affected the results. While no indications suggest that selection procedures played a major role, any possible impact on net demand was likely positive. For example, school administrators may have sent letters only to students whose parents they presumed would most likely buy a net. Nonetheless, similar findings regarding price elasticity (Madagascar) and overall propensity to buy (India) from previous randomized trials on net demand suggest that selection bias did not greatly affect the results here [28, 34].

## Conclusions

This study finds generally robust demand for bed nets among a sample of 800 Tanzanian households. The results stem from a non-hypothetical choice experiment where participants choose to buy or not buy a net from among two nets of various prices, sizes, shapes, and insecticide treatment options. The households’ socio-economic status does not affect net demand. However, a key factor affecting demand is the household’s current net ownership: when there are insufficient nets available to cover household members, which is more often true in rural areas, households show a greater likelihood of buying a net. Price does not exert a large impact on demand, with price elasticities under −0.50, and marginal WTP for various attributes such as large size, square shape or insecticide treatment varies from US$0.75–2. The results imply that the net manufacturers and retailers can successfully market nets to the public by focusing on these attributes, and that governments and policy makers can use this as a viable option to increase access to ITNs in conjunction with other public sector distribution channels.

## Abbreviations

DCE: discrete choice experiment
ITN: insecticide-treated net
LLIN: long-lasting insecticidal net
TSH: Tanzanian shillings
WHO: World Health Organization
WTP: willingness-to-pay
